# Assessment of health equity consideration in masking/PPE policies to contain COVID-19 using PROGRESS-plus framework: a systematic review

**DOI:** 10.1186/s12889-021-11688-7

**Published:** 2021-09-16

**Authors:** Anindit Chhibber, Aditi Kharat, Dylan Kneale, Vivian Welch, Mukdarut Bangpan, Nathorn Chaiyakunapruk

**Affiliations:** 1 School of Pharmacy, University of Utah, Salt Lake City, UT USA; 2 The Evidence for Policy and Practice Information and Co-ordinating Centre (EPPI-Centre), UCL Social Research Institute, University College London, London, UK; 3 Bruyere Research Institute, Ottawa, Canada; 4 School of Epidemiology and Public Health, University of Ottawa, Ottawa, Ontario Canada; 5 School of Pharmacy, Monash University, Subang Jaya, Malaysia

## Abstract

**Introduction:**

There is increasing evidence that COVID-19 has unmasked the true magnitude of health inequity worldwide. Policies and guidance for containing the infection and reducing the COVID-19 related deaths have proven to be effective, however the extent to which health inequity factors were considered in these policies is rather unknown. The aim of this study is to measure the extent to which COVID-19 related policies reflect equity considerations by focusing on the global policy landscape around wearing masks and personal protection equipment (PPE).

**Methods:**

A systematic search for published documents on COVID-19 and masks/PPE was conducted across six databases: PubMed, EMBASE, CINAHL, ERIC, ASSIA and Psycinfo. Reviews, policy documents, briefs related to COVID-19 and masks/PPE were included in the review. To assess the extent of incorporation of equity in the policy documents, a guidance framework known as ‘PROGRESS-Plus’: Place of residence, Race/ethnicity, Occupation, Gender/sex, Religion, Education, Socioeconomic status, Social capital, Plus (age, disability etc.) was utilized.

**Results:**

This review included 212 policy documents. Out of 212 policy documents, 190 policy documents (89.62%) included at least one PROGRESS-plus component. Most of the policy documents (*n* = 163, 85.79%) focused on “occupation” component of the PROGRESS-plus followed by personal characteristics associated with discrimination (*n* = 4;2.11%), place of residence (*n* = 2;1.05%) and education (*n* = 1;0.53%). Subgroup analysis revealed that most of the policy documents (*n* = 176, 83.01%) were focused on “workers” such as healthcare workers, mortuary workers, school workers, transportation workers, essential workers etc. Of the remaining policy documents, most were targeted towards whole population (*n* = 30; 14.15%). Contrary to “worker focused” policy documents, most of the ‘whole population focused’ policy documents didn’t have a PROGRESS-plus equity component rendering them equity limiting for the society.

**Conclusion:**

Our review highlights even if policies considered health inequity during the design/implementation, this consideration was often one dimensional in nature. In addition, population wide policies should be carefully designed and implemented after identifying relevant equity related barriers in order to produce better outcomes for the whole society.

**Supplementary Information:**

The online version contains supplementary material available at 10.1186/s12889-021-11688-7.

## Introduction

COVID-19 is an infectious disease spread by the novel severe acute respiratory syndrome coronavirus 2 (SARS-CoV-2), that first reported in Wuhan, China in 2019 [1]. At the start of the pandemic, SARS-CoV-2 was initially thought to spread mainly through close person to person contact because of production of respiratory droplets formed through a sneeze or cough of an infected person. Later evidence demonstrates that the virus also infects through airborne transmission routes when an infected person ‘exhales, speaks, shouts, sings, sneezes, or coughs’ [2]. SARS-CoV-2 viral particles range from larger respiratory droplets to smaller aerosols [3], making the wearing of high quality and well-fitting masks indoors of particular importance in reducing transmission among the public, and the wearing of additional protective equipment important in reducing transmission among frontline healthcare staff [2]. As of January 5th 2021, 86.2 million cases of COVID-19 have been identified across 218 countries and territories resulting in 1.87 million deaths across the globe [4]. Among the reported cases, 20,551,680 confirmed cases and 349,890 deaths were reported in the USA and (at the time of writing, January 2021) it has become the country with the greatest number of infection and deaths due to COVID-19 [4]. Contrary to US, countries like New Zealand and Vietnam reported 2181 and 1494 cases of COVID-19 resulting into 25 and 35 deaths only respectively [4].

This dire situation demands that everyone has a fair and just opportunity to be as healthy as possible but there is increasing evidence that COVID-19 has unmasked the true magnitude of health inequity worldwide. For example, in US, American Indians or Alaskan natives, African Americans and Hispanic or Latino people have 1.8 times, 1.4 times and 1.7 times higher rate of COVID-19 cases; have 4.0 times, 3.7 times and 4.1 times higher rate of COVID-19 related hospitalizations and 2.6 times, 2.8 times and 2.8 times higher rate of COVID-19 related deaths respectively when compared to their Caucasian counterparts [5]. In addition, elderly people [6], healthcare and frontline workers [7] are at an elevated risk of acquiring COVID-19 and developing severe COVID-19 related outcomes. Thus, to achieve health equity, healthcare policies around COVID-19 should ideally address these inequities so that everyone has a fair opportunity to be as healthy as possible, and the whole society benefits.

Policies and guidance for containing the infection and reducing the COVID-19 related deaths are complex and rapidly evolving. Since no pharmaceutical agents were known to be safe and effective at preventing or treating COVID-19 until recently, only non-pharmaceutical interventions were relied upon for reducing the burden of COVID-19 during the first wave(s) of the pandemic in 2020 [8–11]. These measures aimed to reduce disease transmission both locally and globally and included bans on public gatherings, compulsory stay-at-home policies, mandating closures of schools and nonessential businesses, face mask ordinances, quarantine and cordon sanitaire, among others. The effectiveness of these interventions to reduce COVID-19 transmission has been demonstrated [8–11], however the extent to which health inequity factors were considered in these policies is unknown.

Thus, the aim of this study is to measure the extent to which national, regional, institutional and organizational policies reflect equity considerations by focusing on the global policy landscape around wearing masks and personal protection equipment (PPE). Masks/PPE policies were chosen as area of target as these policies affect more people when compared to other interventional policies such as school closure policies, stay at home policies etc. Equity would be assessed using previously developed Cochrane PROGRESS-Plus equity framework. PROGRESS-plus defines the characteristics that stratify health opportunities and outcomes that can mark inequalities. Current masking/PPE policies implemented across world would ideally be expected to reflect equity considerations in order to effectively manage the spread of the disease and to reduce adverse outcomes, although the extent to which this is the case is uncertain. For example, we may expect equity of access to masks/PPE to be considered when policy-makers are designing policies around the need to wear a mask, or for equity to be considered when undertaking health promotion activities in promoting the importance of mask wearing. The understanding of health equity consideration in masking/PPE policies across globe to contain COVID-19 using PROGRESS-Plus framework will help to achieve a better and more sustainable future for all past the barrier of inequity.

## Methods

This systematic review was done in accordance with Preferred Reporting Items for Systematic Reviews and Meta-Analyses (PRISMA) guidelines [12–19] (Table S1), following a predetermined published protocol (PROSPERO registration: CRD42021231497).

### Search strategy and data sources

We performed a comprehensive search in six electronic databases – PubMed, EMBASE, CINAHL, ERIC, ASSIA and Psycinfo. The search strategy was based on a broad combined search string for COVID-19 and ‘masks or PPE’(Table S2). The searches were conducted to retrieve potentially relevant publications from January 1st, 2020 to July 1st, 2020. Additional literature was identified by searching the reference list of the identified eligible documents.

### Inclusion criteria

All identified documents were evaluated for the inclusion based on the following criteria: (1) documents should be related to COVID-19 and (2) documents should have masks or PPE as an intervention/strategy to mitigate COVID-19. Selection criteria were not limited to any specific kind of study design or type of publication thus allowing reviews, policy documents, or research briefs to be included in the systematic review. Systematic reviews focusing on a number of policies were excluded to decrease repetition among the included documents. Selection criteria were not limited to any specific language thus minimizing language bias. Two reviewers (AC and AK) independently undertook the screening of the records (by title and/or abstract) for eligibility and a third reviewer (NC) mediated if contradiction to arrive at an accord occurred. Full text of eligible papers after the first screening was reviewed to confirm that the articles met the inclusion and exclusion criteria. Similar to title/abstract screening, full text screening was also done by two reviewers independently and a third reviewer mediated if contradiction of an accord occurred.

### Data extraction

A customized data extraction sheet was constructed to extract relevant data from all documents meeting our inclusion criteria. The data abstracted included: author(s), publication year, the geographical location of data collection, study design, setting, target population, implementation level of policy, equity incorporation, equity component, strength of evidence and key findings. Similar to the screening process, data extraction was conducted by two reviewers (AC and AK) and any contradiction was resolved by a third reviewer (NC).

### Assessment of incorporation of equity

To assess the extent of incorporation of equity in policies, we utilized a guidance framework known as ‘PROGRESS-Plus’ [20, 21]. This assessment was conducted in order to analyze the extent to which equity has been incorporated in PPE or masking policies and implementation of these policies around the globe. PROGRESS-plus equity framework is aimed at warranting the consideration of various health inequity inducing factors such as place of residence, race/ethnicity, occupation, gender, religion, education, socioeconomic status and personal characteristics when devising policies and/or guidelines. Additional details about the framework can be found elsewhere [20, 21]. The data from included documents were analyzed to determine whether a study has considered equity component. If the included policy had any of the above-mentioned PROGRESS-Plus components it was determined that the study had incorporated an equity component. Where a measure was classifiable under more than one PROGRESS-Plus factor (e.g. an indicator of employment status is relevant to ‘occupation’ but also to ‘socio-economic status’ (SES)), we included it under the factor deemed more appropriate. It is important to note that utilization of PROGRESS-plus framework for this research is context specific and findings are limited to COVID-19 for most part. For instance, population types such as healthcare workers, essential workers are not usually considered vulnerable in society but in the context of COVID-19, increased risk of transmission of COVID-19 puts them at a disadvantage compared to the general population. Thus, findings of the research work should be interpreted within aforementioned scope.

Second, the provision and strength of the rationale to support inclusion of “PROGRESS-plus” factor in a policy was analyzed. The aim of this analysis was to ascertain whether policies have included an equity component following the empirical evidence or not. The documents were divided into two groups: explicit rationale or implicit rationale. If a study/policy provided the empirical evidence for inclusion of PROGRESS-plus component, the study was deemed to have an ‘explicit rationale’. The study/policy was deemed to have an ‘implicit rationale’ if no empirical evidence was provided for the inclusion of PROGRESS-plus component.

Third, “indication level of equity” in documents was assessed. The aim of this analysis was to ascertain whether “equitable health” was a primary factor while framing the policies (defined as high level) or documents were focused on a certain group or subpopulation thus having “equitable health” as a latent factor (defined as low level). The major difference between these two categories is that ‘high level’ documents acknowledge ‘health equity’ related issues, as opposed to ‘low level’ documents which fail to mention them. For example, if a study/policy was targeted at use of face masks among healthcare workers without any mention of ‘equity’, ‘inequity’, ‘health disparities’ in aims, objectives or discussion, it was deemed to be ‘low level’. On the contrary if a study/policy was targeted at use of face masks among healthcare workers with the mention of ‘equity’, ‘inequity’, ‘health disparities’ in aims, objectives or discussion, it was deemed to be ‘high level’.

### Data analysis

A narrative synthesis of data was conducted as most of the included documents were policies and lacked statistical results. We presented the data in the two distinct sections. The first section aimed at outlining the included policies in the review and second section aimed at explaining the equity component in the eligible documents. The results are presented in a descriptive manner using frequencies, percentages and pie charts. EPPI-reviewer [22] was utilized for the management and analysis of the data . In addition, due to subjective nature of the extracted policy documents, no ‘strength of evidence’ analysis was performed.

## Results

**Fig. 1:**
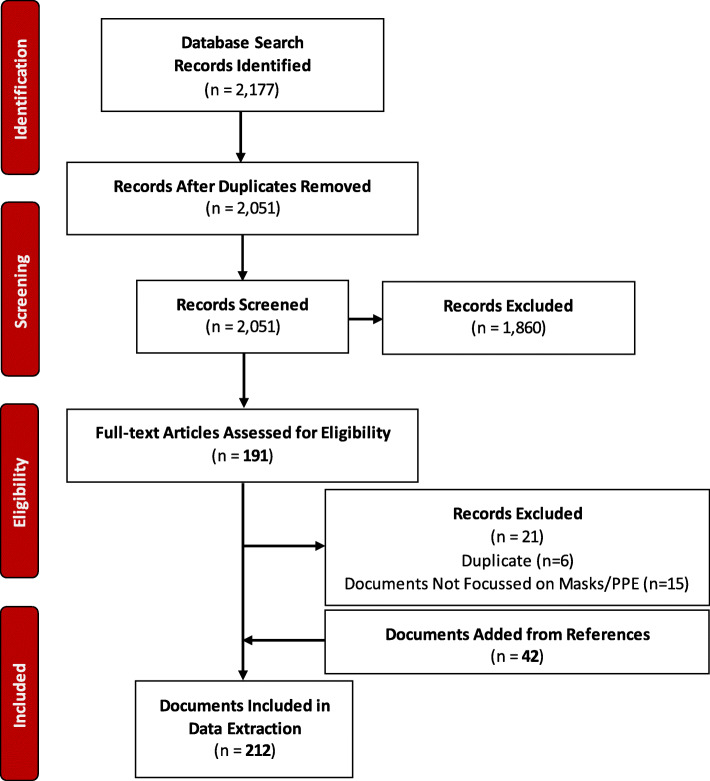
PRISMA Flow for Selection of the Included Policy Documents

The search strategy yielded 2177 articles that were focused on COVID-19. Out of these 2177 articles 125 duplicates were removed. Of the remaining 2051 articles only 191 met the inclusion criteria and were retrieved to be reviewed in full-text. In addition, 42 policy documents retrieved from references of these included articles were added to be reviewed in full text. During the full-text screening, further 21 articles were excluded due to following reasons; duplicate (*n* = 6), and irrelevant/non mask/PPE policy (*n* = 15). This resulted in a total of 212 [23–234] relevant articles to be included in this systematic review (Fig. 1). The screening yielded two types of documents: original policies and policy recommendations. Original policies were the documents issued by government agencies such as CDC, WHO or alike whereas policy recommendations consisted of documents that were not policies themselves but contained different recommendations for the policy. Both original policies and policy recommendations will be referred to as “policy documents” from here on in the review.

### General study characteristics

**Fig. 2:**
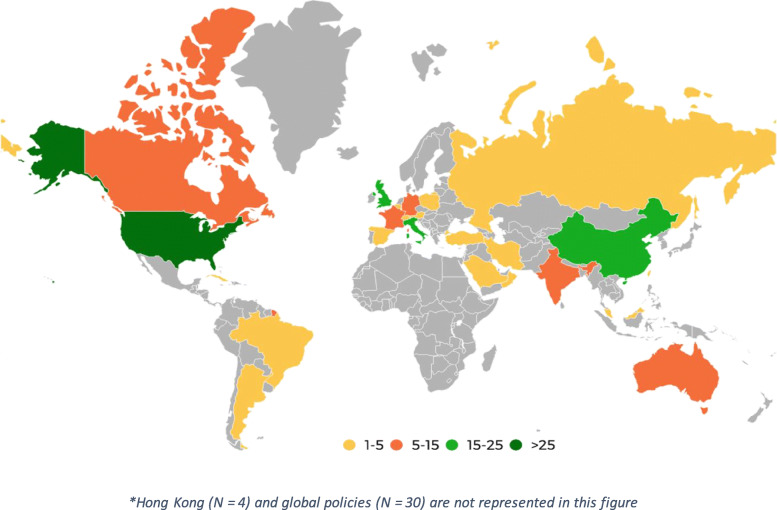
Number of Included Policy Documents Categorized by Nation*

General characteristics of the included policy documents are summarized in Table 1. Masks/PPE as a mitigation strategy against COVID-19 was implemented across different regions ranging from USA to Australia to Russia (Table 1 and Fig. 2). There were no policy documents identified from Eastern Europe, Africa and Middle Eastern regions in this review. There were 29 policy documents [25, 34, 41, 59, 75, 125, 126, 133, 134, 140, 141, 146, 155, 157, 166, 177, 178, 208–219] that were not targeted towards a specific nation or region but were global in nature.

**Table 1:** Characteristics of The Included Policy Documents

Author and Year	Title	Country	HIC/LMIC	Implementation	Target
Abramowicz J S [25]	ISUOG Safety Committee Position Statement on Use Of Personal Protective Equipment And Hazard Mitigation In Relation To Sars-Cov-2 For Practitioners Undertaking Obstetric And Gynaecological Ultrasound	Global	HIC and LMIC	Institutional	Healthcare Workers
Ağalar C [27]	Protective Measures for Covid-19 For Healthcare Providers and Laboratory Personnel	Turkey	LMIC	Institutional	Healthcare Workers
Amatya S [28]	Management of New-borns Exposed To Mothers With Confirmed Or Suspected Covid-19	USA	HIC	Institutional	Healthcare Workers
American Academy of Ophthalmology [29]	Important coronavirus updates for ophthalmologists	USA	HIC	Institutional	Patients and healthcare workers
American Academy of Paediatrics [31]	Initial guidance: Management of infants born to mothers with COVID-19	USA	HIC	Institutional	Patients and healthcare workers
American College of Cardiology [32]	Catheterization Laboratory Considerations During the Coronavirus (COVID-19) Pandemic: From the ACC’s Interventional Council and SCAI	USA	HIC	Institutional	Healthcare Workers
American Geriatrics Society [33]	American Geriatrics Society (AGS) Policy Brief: COVID-19 and Assisted Living Facilities	USA	HIC	National	Population Wide
AOCMF [34]	Recommendations on best practices for Maxillofacial Procedures during COVID 19 Pandemic	Global	HIC and LMIC	Institutional	Patients and healthcare workers
Ashari M A [35]	Strategies for radiology departments in handling the COVID-19 pandemic	Malaysia	LMIC	Institutional	Healthcare Workers
Ayan A [36]	Guide for nuclear medicine applications during the covid-19 outbreak	Turkey	LMIC	Institutional	Healthcare Workers
Bann D V [37]	Impact of coronavirus (COVID-19) on otolaryngologic surgery: Brief commentary	USA	HIC	Institutional	Healthcare Workers
Bann Darrin V [38]	Best Practice Recommendations for Pediatric Otolaryngology during the COVID-19 Pandemic	USA	HIC	Institutional	Healthcare Workers
Bein B [39]	SARS-CoV-2/COVID-19: Evidence-Based Recommendations on Diagnosis and Therapy	Germany	HIC	National	Healthcare Workers
Bianco F [40]	Preventing transmission among operating room staff during COVID-19 pandemic: the role of the Aerosol Box and other personal protective equipment	Italy	HIC	Institutional	Healthcare Workers
Bikson M [41]	Guidelines for TMS/tES clinical services and research through the COVID-19 pandemi	Global	HIC and LMIC	Global	Healthcare Workers
Boccalatte L A [42]	Brief guideline for the prevention of COVID-19 infection in head and neck and otolaryngology surgeons	Argentina	HIC	Institutional	Healthcare Workers
National Health Commission of the People’s Republic of China [23]	Diagnosis and Treatment Protocol for Novel Coronavirus Pneumonia	China	LMIC	National	Healthcare Workers
CAG [43]	COVID-19: Advice from the Canadian Association of Gastroenterology for Endoscopy Facilities	Canada	HIC	Institutional	Patients and healthcare workers
Capanna F [45]	Preparing An Obstetric Unit In The Heart Of The Epidemic Strike Of Covid-19: Quick Reorganization Tips	Switzerland, Spain, Russia, Austria, Italy	HIC	Institutional	Healthcare Workers
Cardinale F [46]	Consensus Statement Of The Italian Society Of Pediatric Allergy And Immunology For The Pragmatic Management Of Children And Adolescents With Allergic Or Immunological Diseases During The Covid-19 Pandemic	Italy	HIC	Institutional	Healthcare Workers
Carneiro A [47]	Impact of the COVID-19 Pandemic on the Urologist’s clinical practice in Brazil: a management guideline proposal for low- and middle-income countries during the crisis period	Brazil	LMIC	National	Healthcare Workers
Casiraghi A [48]	Operational Strategies of A Trauma Hub In Early Coronavirus Disease 2019 Pandemic	Italy	HIC	Institutional	Healthcare Workers
Castelnuovo P [49]	Skull Base Surgery During the Covid-19 Pandemic: The Italian Skull Base Society Recommendations	Italy	HIC	Institutional	Healthcare Workers
CDC [50]	Coronavirus Disease 2019 (COVID-19). Frequently Asked Questions about Personal Protective Equipment.	USA	HIC	National	Population Wide
CDC [51]	Use Personal Protective Equipment (PPE) When Caring for Patients with Confirmed or Suspected COVID-19	USA	HIC	Institutional	Healthcare Workers
CDC [52]	Use of Masks to Help Slow the Spread of COVID-19	USA	HIC	National	Population Wide
CDC [53]	Criteria for Return to Work for Healthcare Personnel with SARS-CoV-2 Infection (Interim Guidance)	USA	HIC	Institutional	Healthcare Workers
CDC [54]	Interim Infection Prevention and Control Recommendations for Healthcare Personnel During the Coronavirus Disease 2019 (COVID-19) Pandemic	USA	HIC	Global	Healthcare Workers
CDC [55]	Standard Precautions for All Patient Care	USA	HIC	National	Patients
CDC [56]	Interim Clinical Guidance for Management of Patients with Confirmed Coronavirus Disease (COVID-19)	USA	HIC	National	Patients
CDC [57]	Interim Guidance on Management of Coronavirus Disease 2019 (COVID-19) in Correctional and Detention Facilities	USA	HIC	National	Healthcare Workers
Chahar P [58]	Airway Management Considerations In Patients With Covid-19	USA	HIC	Institutional	Patients and healthcare workers
Chan T K [59]	Universal Masking For Covid-19: Evidence, Ethics And Recommendations	Global	HIC and LMIC	Global	Population Wide
Chandra A [60]	Personal protective equipment (PPE) for vitreoretinal surgery during COVID-19	UK	HIC	Global	Healthcare Workers
Chavez S [61]	Coronavirus Disease (Covid-19): A Primer For Emergency Physicians	USA	HIC	Institutional	Healthcare Workers
Chawla D [62]	Perinatal-Neonatal Management Of Covid-19 Infection - Guidelines Of The Federation Of Obstetric And Gynaecological Societies Of India (FOGSI), National Neonatology Forum Of India (NNF), And Indian Academy Of Paediatrics (IAP)	India	LMIC	Institutional	Healthcare Workers
Chen W [64]	To Protect Healthcare Workers Better, To Save More Lives	China	LMIC	Institutional	Healthcare Workers
Chen X C [65]	Preventive and Control Measures for the ﻿Coronavirus Pandemic in Clinical Dentistry	China	LMIC	Institutional	Healthcare Workers
Chersich M F [66]	Covid-19 In Africa: Care And Protection For Frontline Healthcare Workers	Africa	HIC and LMIC	Institutional	Healthcare Workers
Ciavattini A [71]	Expert Consensus From The Italian Society For Colposcopy And Cervico-Vaginal Pathology (SICPCV) For Colposcopy And Outpatient Surgery Of The Lower Genital Tract During The Covid-19 Pandemic	Italy	HIC	Institutional	Healthcare Workers
CMS [72]	CMS Issues Recommendations to Re-Open Health Care Systems in Areas with Low Incidence of COVID-19	USA	HIC	National	Healthcare Workers
Coccolini Federico [73]	Surgery in COVID-19 patients: operational directives	Italy	HIC	Institutional	Healthcare Workers
Communicable Disease Network Australia [195]	Novel Coronavirus 2019 (2019-nCoV) Infection: Part II - Respiratory Support in the Pediatric Intensive Care Unit in Resource-limited Settings	Australia	HIC	National	Patients and healthcare workers
Copland M [74]	Canadian Society Of Nephrology Covid-19 Rapid Response Team Home Dialysis Recommendations	Canada	HIC	Institutional	Patients and healthcare workers
Corburn J [75]	Slum Health: Arresting COVID-19 and Improving Well-Being in Urban Informal Settlements	Global	HIC and LMIC	Global	Population Wide
Couloigner V [76]	Covid-19 And ENT Surgery	France	HIC	Institutional	Healthcare Workers
Crosby D L [77]	Evidence-Based Guidelines for Management of Head and Neck Mucosal Malignancies during the COVID-19 Pandemic	USA	HIC	Institutional	Healthcare Workers
Cui C [78]	Approaching Otolaryngology Patients During the COVID-19 Pandemic	China	LMIC	Institutional	Healthcare Workers
Curigliano G [79]	How To Guarantee The Best Of Care To Patients With Cancer During The Covid-19 Epidemic: The Italian Experience	Italy	HIC	National	Healthcare Workers
D’Aguanno V [80]	Clinical Recommendations for Epistaxis Management During the COVID-19 Pandemic	Italy	HIC	Institutional	Healthcare Workers
Davenport M S [81]	ACR Statement On Safe Resumption Of Routine Radiology Care During The Coronavirus Disease 2019 (Covid-19) Pandemic	USA	HIC	Institutional	Patients and healthcare workers
Day A T [82]	Head And Neck Oncology During The Covid-19 Pandemic: Reconsidering Traditional Treatment Paradigms In Light Of New Surgical And Other Multilevel Risks	USA	HIC	Institutional	Patients and healthcare workers
Deepthi Ramamurthy [83]	Personal Protective Equipments (PPE) -- Prerequisites, Rationale and Challenges during COVID 19 Pandemic	India	LMIC	National	Healthcare Workers
Demirbilek Y [84]	Covid-19 Control, Example Of Ministry Of Health Of Turkey	Turkey	LMIC	National	Population Wide
Desai U [85]	Guidance For Resumption Of Routine Electrodiagnostic Testing During The Covid-19 Pandemic	USA	HIC	Institutional	Patients and healthcare workers
DeSerres J J [86]	Best Practice Guidelines For The Management Of Acute Craniomaxillofacial Trauma During The Covid-19 Pandemic	Canada	HIC	Institutional	Healthcare Workers
Dexter F [88]	Perioperative COVID-19 Defense: An Evidence-Based Approach for Optimization of Infection Control and Operating Room Management	USA	HIC	Institutional	Healthcare Workers
DGP [87]	Recommendations for treatment of patients with COVID-19 from the palliative care perspective V2.0	Germany	HIC	National	Healthcare Workers
Di Saverio [89]	Coronavirus Pandemic And Colorectal Surgery: Practical Advice Based On The Italian Experience	Italy	HIC	Institutional	Population Wide
Dockery D M [91]	The Ocular Manifestations And Transmission Of Covid-19: Recommendations For Prevention	USA	HIC	Institutional	Healthcare Workers
Dyal J W [92]	COVID-19 Among Workers in Meat and Poultry Processing Facilities - 19 States, April 2020	USA	HIC	National	Meat/poultry workers
Elli L [93]	Endoscopy During The Covid-19 Outbreak: Experience And Recommendations From A Single Center In A High-Incidence Scenario	Italy	HIC	Institutional	Healthcare Workers
Esposito S [94]	To mask or not to mask children to overcome COVID-19	Italy	HIC	National	Patients
Esposito S [95]	Universal use of face masks for success against COVID-19: evidence and implications for prevention policies	Italy & China	HIC and LMIC	Global	Population Wide
European Centre for Disease Prevention and Control [98]	Infection prevention and control and preparedness for COVID-19 in healthcare settings - fourth update	Europe	HIC and LMIC	National	Healthcare Workers
European Centre for Disease Prevention and Control (ECDC) [96]	Infection Prevention and Control For the Care of Patients With 2019-nCoV in Healthcare Setting	Europe	HIC and LMIC	Institutional	Healthcare Workers
European Centre for Disease Prevention and Control [97]	Infection prevention and control for the care of patients with 2019-nCoV in healthcare settings	Europe	HIC and LMIC	National	Healthcare Workers and Hospital Administration
Evidence-Based Medicine Chapter of China [128]	A rapid advice guideline for the diagnosis and treatment of 2019 novel coronavirus (2019-nCoV) infected pneumonia (standard version)	China	LMIC	Institutional	Patients and healthcare workers
Fananapazir G [99]	Reorganizing Cross-Sectional Interventional Procedures Practice During the Coronavirus Disease (COVID-19) Pandemic	USA	HIC	Institutional	Healthcare Workers
Fawley N [100]	Procedural sedation in the COVID-19 era	USA	HIC	Institutional	Patients
Fillingham Y A [101]	Personal Protective Equipment: Current Best Practices for Orthopedic Teams	USA	HIC	Institutional	Healthcare Workers
Forrester J D [102]	Precautions for Operating Room Team Members During the COVID-19 Pandemic	USA	HIC	Institutional	Healthcare Workers
Francis N [103]	SAGES and EAES recommendations for minimally invasive surgery during COVID-19 pandemic	USA, UK, Italy, Spain	HIC	Institutional	Healthcare Workers
Friese C R [104]	Respiratory Protection Considerations For Healthcare Workers During The Covid-19 Pandemic	USA	HIC	Institutional	Healthcare Workers
Furfaro F [105]	SFED recommendations for IBD endoscopy during COVID-19 pandemic: Italian and French experience	Italy and France	HIC	Institutional	Healthcare Workers
Gackowski A [106]	Echocardiography During The Coronavirus Disease 2019 (Covid-19) Pandemic: Expert Opinion Of The Working Group On Echocardiography Of The Polish Cardiac Society	Poland	HIC	Institutional	Patients and healthcare workers
Galloro G [107]	Safety in digestive endoscopy procedures in the covid era recommendations in progres of the italian society of digestive endoscopy	Italy	HIC	Institutional	Healthcare Workers
Gemicioğlu Bilun [108]	Turkish Thoracic Society Experts Consensus Report: Recommendations for Pulmonary Function Tests During and After COVID 19 Pandemic	Turkey	LMIC	Institutional	Healthcare Workers
George M [109]	Proposal of a timing strategy for cholesteatoma surgery during the COVID-19 pandemic	Oman, Italy, India	HIC and LMIC	Institutional	Healthcare Workers
Goh Ken Junyang [110]	Preparing your intensive care unit for the COVID-19 pandemic: practical considerations and strategies	Singapore	HIC	Institutional	Healthcare Workers
Gorry C [111]	Covid-19 Case Detection: Cuba’S Active Screening Approach	Cuba	LMIC	National	Population Wide
Gosling A F [112]	Perioperative Considerations for Tracheostomies In The Era Of Covid-19	USA	HIC	Institutional	Healthcare Workers
Government of Canada [113]	Infection prevention and control for COVID-19: Second interim guidance for acute healthcare settings	Canada	HIC	Institutional	Healthcare Workers
Gupta Arti [114]	Managing a COVID 19 patient at different health care and field level settings	India	LMIC	Institutional	Population Wide
Gupta P [115]	Neurosurgery and Neurology Practices during the Novel COVID-19 Pandemic: A Consensus Statement from India	India	LMIC	Institutional	Healthcare Workers
Ha J F [116]	The covid-19 pandemic, personal protective equipment, and respirator: a narrative review	Australia	HIC	Global	Healthcare Workers
Haines S [117]	Practical Considerations When Performing Neurodiagnostic Studies on Patients with COVID-19 and Other Highly Virulent Diseases	USA	HIC	Institutional	Healthcare Workers
Han G [118]	Possibly Critical Role Of Wearing Masks In General Population In Controlling Covid-19	China	LMIC	Global	Population Wide
Healthcare Purchasing News [119]	CDC Updates Recommendations For Healthcare Supply Of PPE	USA	HIC	National	Healthcare Workers
Heldwein F L [120]	A Systematic Review on Guidelines and Recommendations for Urology Standard of Care During the COVID-19 Pandemic	UK, USA, China	HIC and LMIC	Institutional	Healthcare Workers
Higginson Ray [121]	Paramedic Use Of PPE And Testing During The COVID-19 Pandemic	UK	HIC	Institutional	Healthcare Workers
Higginson Ray [122]	Personal protective equipment and testing during the COVID-19 pandemic	UK	HIC	Institutional	Healthcare Workers
Hirschmann M T [123]	COVID-19 coronavirus: recommended personal protective equipment for the orthopaedic and trauma surgeon	Austria, Luxembourg, Switzerland, Germany, UK	HIC	Institutional	Healthcare Workers
Hsieh T Y [124]	A Guide To Facial Trauma Triage And Precautions In The Covid-19 Pandemic	USA	HIC	Institutional	Healthcare Workers
Hu-Friedy [125]	Best Practices For Hand Hygiene And Face Mask Use	Global	HIC and LMIC	Institutional	Healthcare Workers
Iacucci M [126]	Endoscopy In Inflammatory Bowel Diseases During The Covid-19 Pandemic And Post-Pandemic Period	Global	HIC and LMIC	Institutional	Healthcare Workers
Islam M S [127]	Examining the current intelligence on COVID-19 and infection prevention and control strategies in health settings: A global analysis	Australia	HIC	Global	Healthcare Workers
Jung F [129]	How we should respond to the Coronavirus SARS-CoV-2 outbreak: A German perspective	Germany	HIC	National	Population Wide
Kabesch M [130]	Successful Containment Of Covid-19 Outbreak In A Large Maternity And Perinatal Center While Continuing Clinical Service	Germany	HIC	Institutional	Healthcare Workers
Kligerman M P [131]	Managing Head And Neck Cancer Patients With Tracheostomy Or Laryngectomy During The Covid-19 Pandemic	USA and China	HIC and LMIC	Institutional	Patients and healthcare workers
Kluge S [132]	German recommendations for critically ill patients with COVID-19	Germany	HIC	Institutional	Patients
Korobelnik J F [133]	Guidance for anti-VEGF intravitreal injections during the COVID-19 pandemic	Global	HIC and LMIC	Institutional	Healthcare Workers
Kowalski L P [134]	Covid-19 Pandemic: Effects And Evidence-Based Recommendations For Otolaryngology And Head And Neck Surgery Practice	Global	HIC and LMIC	Institutional	Healthcare Workers
Lammers Marc J. W [135]	Guidance For Otolaryngology Health Care Workers Performing Aerosol Generating Medical Procedures During The Covid-19 Pandemic	Canada	HIC	Institutional	Healthcare Workers
Lavinsky J [136]	An Update On Covid-19 For The Otorhinolaryngologist – A Brazilian Association Of Otolaryngology And Cervicofacial Surgery (Aborl-Ccf) Position Statement	Brazil	LMIC	Institutional	Healthcare Workers
Leboulanger N [137]	Covid-19 And Ent Pediatric Otolaryngology During The Covid-19 Pandemic. Guidelines Of The French Association Of Pediatric Otorhinolaryngology (Afop) And French Society Of Otorhinolaryngology (Sforl)	France	HIC	Institutional	Patients and healthcare workers
Lescanne E [139]	Best practice recommendations: ENT consultations during the COVID-19 pandemic	France	HIC	Institutional	Healthcare Workers
Leung C C [140]	Mask Wearing To Complement Social Distancing And Save Lives During Covid-19	Global	HIC and LMIC	Global	Population Wide
Leung Chi Chiu [141]	Mass Masking In The Covid-19 Epidemic: People Need Guidance	Global	HIC and LMIC	Global	Population Wide
Li Zhijie [142]	How Ophthalmologists should understand and respond to the current epidemic of novel coronavirus pneumonia	China	LMIC	Institutional	Healthcare Workers
Lie S A [143]	Practical considerations for performing regional anesthesia: lessons learned from the COVID-19 pandemic	Singapore	HIC	Institutional	Healthcare Workers
Lim L W [144]	Sustainable Practice Of Ophthalmology During Covid-19: Challenges And Solutions	Singapore	HIC	Institutional	Healthcare Workers
Lockhart S L [145]	Personal protective equipment (PPE) for both anesthesiologists and other airway managers: principles and practice during the COVID-19 pandemic	Canada	HIC	Institutional	Healthcare Workers
Ludovico G [138]	Hospital care in Departments defined as Covid-free: A proposal for a safe hospitalization protecting healthcare professionals and patients not affected by Covid 19	Italy	HIC	Institutional	Healthcare Workers
MacIntyre C Raina [172]	Community Universal Face Mask Use During The Covid 19 Pandemic-From Households To Travellers And Public Spaces	USA	HIC	Global	Population Wide
Matava C T [146]	Pediatric Airway Management In Covid-19 Patients - Consensus Guidelines From The Society For Pediatric Anesthesia’S Pediatric Difficult Intubation Collaborative And The Canadian Pediatric Anesthesia Society	Global	HIC and LMIC	Institutional	Healthcare Workers
Mattei A [147]	Guidelines of clinical practice for the management of swallowing disorders and recent dysphonia in the context of the COVID-19 pandemic	France	HIC	Institutional	Healthcare Workers
McGrath B A [148]	Multidisciplinary Guidance For Safe Tracheostomy Care During The Covid-19 Pandemic: The Nhs National Patient Safety Improvement Programme (Natpatsip)	UK	HIC	Institutional	Healthcare Workers
Micali G [149]	The Italian dermatologic community facing COVID-19 pandemic: recommendation from the Italian society of dermatology and venereology	Italy	HIC	Institutional	Healthcare Workers
Milad Abdi [24]	Coronavirus disease 2019 (COVID-19) outbreak in Iran: actions and problems	Iran	LMIC	National	Population Wide
Mujoomdar A [150]	The Canadian Association for Interventional Radiology (CAIR) and Canadian Association of Radiologists (CAR) Guidelines for Interventional Radiology Procedures for Patients With Suspected or Confirmed COVID-19	Canada	HIC	Institutional	Healthcare Workers
Mupparapu Mel [151]	Dental Practitioners’ Role In The Assessment And Containment Of Coronavirus Disease (Covid-19): Evolving Recommendations From The Centers For Disease Control	USA	HIC	Institutional	Patients and healthcare workers
Mytrang H. Do [90]	Recommendations For Personal Protective Equipment And Smoke Evacuation For Dermatologic Surgeries Amid The Covid-19 Crisis	USA	HIC	Institutional	Healthcare Workers
Ng J J [152]	Experience from a Singapore tertiary hospital with restructuring a vascular surgery practice in response to national and institutional policies during the COVID-19 pandemic	Singapore	HIC	Institutional	Healthcare Workers
NICE [153]	COVID-19 rapid guideline: critical care in adults	UK	HIC	Institutional	Patients and healthcare workers
NICE [154]	COVID-19 rapid guideline: managing symptoms (including at the end of life) in the community	UK	HIC	National	Population Wide
Nolan J P [155]	European Resuscitation Council Covid-19 Guidelines Executive Summary	Global	HIC and LMIC	Institutional	Healthcare Workers
Ortega R [156]	Personal Protective Equipment and Covid-19	USA	HIC	Institutional	Healthcare Workers
Paez D [157]	COVID-19 pandemic: guidance for nuclear medicine departments	Global	HIC and LMIC	Institutional	Healthcare Workers
Palatnik A [158]	Protecting Labor and Delivery Personnel from COVID-19 during the Second Stage of Labor	USA	HIC	Institutional	Healthcare Workers
Pan Lijun [67]	Health protection guideline of enterprises during COVID-19 outbreak	China	LMIC	National	Enterprise employees
Panesar K [159]	Evolution Of Covid-19 Guidelines For University Of Washington Oral And Maxillofacial Surgery Patient Care	USA	HIC	Institutional	Healthcare Workers
Panuganti B A [160]	Procedural Precautions And Personal Protective Equipment During Head And Neck Instrumentation In The Covid-19 Era	China	LMIC	Institutional	Healthcare Workers
Patel V [161]	Cardiac Surgery during the COVID-19 Pandemic: Perioperative Considerations and Triage Recommendations	USA	HIC	Institutional	Healthcare Workers
Patwa A [162]	All India difficult airway association (AIDAA) consensus guidelines for airway management in the operating room during the COVID-19 pandemic	India	LMIC	Institutional	Healthcare Workers
Peditto M [163]	Dentistry during the covid-19 epidemic: An italian workflow for the management of dental practice	Italy	HIC	Institutional	Healthcare Workers
Peraza-Smith George Byron [164]	Imperative for a Safe and Healthy Workplace for Nurses	USA	HIC	Institutional	Healthcare Workers
Perencevich E N [165]	Moving Personal Protective Equipment Into The Community: Face Shields And Containment Of Covid-19	USA	HIC	National	Population Wide
Perkins G D [166]	International Liaison Committee On Resuscitation: Covid-19 Consensus On Science, Treatment Recommendations And Task Force Insights	Global	HIC and LMIC	Institutional	Healthcare Workers
Pezzulla D [167]	Radiotherapy In Southern Italy At The Time Of Covid-19: Options For Radiation Oncology Units	Italy	HIC	Institutional	Healthcare Workers
Public Health England [168]	COVID-19: Infection Prevention and Control Guidance	UK	HIC	Institutional	Patients, healthcare workers and hospital visitors
Public Health England [169]	COVID-19: management of staff and exposed patients or residents in health and social care settings	UK	HIC	National	Healthcare Workers
Public health England [170]	New recommendations for primary and community health care providers in England	UK	HIC	National	Healthcare Workers
Quah Li Juan Joy [171]	Reorganising The Emergency Department To Manage The Covid-19 Outbreak	Singapore	HIC	Institutional	Healthcare Workers
Ramos R F [173]	Recommendations of the Brazilian College of Surgeons for laparoscopic surgery during the COVID-19 pandemic	Brazil	LMIC	Institutional	Healthcare Workers
Randelli P S [174]	Management Of Orthopaedic And Traumatology Patients During The Coronavirus Disease (Covid-19) Pandemic In Northern Italy	Italy	HIC	Institutional	Healthcare Workers
Rochelson Burton [175]	The Care Of Pregnant Women During The Covid-19 Pandemic – Response Of A Large Health System In Metropolitan New York	USA	HIC	Institutional	Patients and healthcare workers
Saadi R A [176]	A Commentary On Safety Precautions For Otologic Surgery During The Covid-19 Pandemic	USA	HIC	Institutional	Healthcare Workers
Saenz L C [177]	Recommendations For The Organization Of Electrophysiology And Cardiac Pacing Services During The Covid-19 Pandemic	Global	HIC and LMIC	Institutional	Healthcare Workers
San-Juan D [178]	Guidance for clinical neurophysiology examination throughout the COVID-19 pandemic. Latin American chapter of the IFCN task force - COVID-19	Global	HIC and LMIC	Institutional	Healthcare Workers
Say D S [179]	Risk stratification and personal protective equipment use in pediatric endoscopy during the coronavirus disease 2019 outbreak: A single-center protocol	USA	HIC	Institutional	Healthcare Workers
Sayburn A [180]	Covid-19: Phe Upgrades Ppe Advice For All Patient Contacts With Risk Of Infection	UK	HIC	Institutional	Healthcare Workers
Schultz P [181]	French consensus regarding precautions during tracheostomy and post-tracheostomy care in the context of COVID-19 pandemic	France	HIC	Institutional	Healthcare Workers
Seely J M [182]	Covid-19: Safe Guidelines For Breast Imaging During The Pandemic	Canada	HIC	Institutional	Patients and healthcare workers
Sengupta S [183]	All India Ophthalmological Society - Indian Journal of Ophthalmology consensus statement on preferred practices during the COVID-19 pandemic	India	LMIC	Institutional	Healthcare Workers
Shaker M S [184]	Covid-19: Pandemic Contingency Planning For The Allergy And Immunology Clinic	USA and Canada	HIC	Institutional	Patients and healthcare workers
Sharma Vandana [185]	Prioritizing vulnerable populations and women on the frontlines: COVID-19 in humanitarian contexts	USA	HIC	Global	Essential Workers
Singh A G [186]	Navigating the impact of COVID-19 on palliative care for head and neck cancer	India	LMIC	Institutional	Healthcare Workers
Singh B [187]	Indian resuscitation council (IRC) suggested guidelines for comprehensive cardiopulmonary life support (CCLS) for suspected or confirmed coronavirus disease (COVID-19) patient	India	LMIC	National	Healthcare Workers
Sobel D [188]	Personal Protective Equipment for Common Urologic Procedures Before and During the United States COVID-19 Pandemic: A Single Institution Study	USA	HIC	Institutional	Healthcare Workers
Soldatova L [189]	Virtual Dysphagia Evaluation: Practical Guidelines for Dysphagia Management in the Context of the COVID-19 Pandemic	USA	HIC	Institutional	Healthcare Workers
Sommer D D [190]	Recommendations From The Cso-Hns Taskforce On Performance Of Tracheotomy During The Covid-19 Pandemic	Canada	HIC	Institutional	Healthcare Workers
Sonali Advani [26]	Universal Masking in Hospitals in the COVID-19 era: Is it Time to consider Shielding?	USA	HIC	National	Healthcare Workers
Sorbello M [191]	The Italian coronavirus disease 2019 outbreak: recommendations from clinical practice	Italy	HIC	National	Population Wide
Spinazzè A [192]	Covid-19 Outbreak In Italy: Protecting Worker Health And The Response Of The Italian Industrial Hygienists Association	Italy	HIC	Institutional	Healthcare Workers, Essential Workers and Population Wide
Steward J E [193]	Urologic Surgery And Covid-19: How The Pandemic Is Changing The Way We Operate	USA	HIC	Institutional	Healthcare Workers
Sunjaya A P [196]	Rationale For Universal Face Masks In Public Against Covid-19	Australia	HIC	National	Population Wide
Tan R M. R [197]	Dynamic Adaptation To Covid-19 In A Singapore Paediatric Emergency Department	Singapore	HIC	Institutional	Healthcare Workers
The American Academy of Otolaryngology–Head and Neck Surgery [30]	AAO Position Statement: Tracheotomy Recommendations During the COVID-19 Pandemic	USA	HIC	National	Healthcare Workers
The American Gastroenterological Association [194]	AGA Rapid Recommendations for Gastrointestinal Procedures During the COVID-19 Pandemic	USA	HIC	National	Healthcare Workers
Thomas John P [198]	Evaluating the national PPE guidance for NHS healthcare workers during the COVID-19 pandemic	UK	HIC	National	Healthcare Workers
Thomas P [199]	Physiotherapy Management For Covid-19 In The Acute Hospital Setting: Clinical Practice Recommendations	Australia	HIC	Institutional	Healthcare Workers
Turkistani K A [200]	Precautions and recommendations for orthodontic settings during the COVID-19 outbreak: A review	Saudi Arabia	HIC	Institutional	Healthcare Workers
Van Gerven L [201]	Personal Protection And Delivery Of Rhinologic And Endoscopic Skull Base Procedures During The Covid-19 Outbreak	Belgium, UK and Spain	HIC	Institutional	Healthcare Workers
Vera C [44]	Transmission risk of SARS-CoV-2 to healthcare workers – observational results of a primary care hospital contact tracing	Switzerland	HIC	Institutional	Healthcare Workers
Wallace M [202]	Public Health Response to COVID-19 Cases in Correctional and Detention Facilities - Louisiana, March–April 2020	USA	HIC	Institutional	Correctional and detention facility Administrators
Walsh C M [203]	Pediatric Endoscopy in the Era of Coronavirus Disease 2019: A North American Society for Pediatric Gastroenterology, Hepatology, and Nutrition Position Paper	USA	HIC	Institutional	Healthcare Workers
Wan Y L [204]	Preparedness and Best Practice in Radiology Department for COVID-19 and Other Future Pandemics of Severe Acute Respiratory Infection	Taiwan and USA	HIC	Institutional	Healthcare Workers
Wang Qiang [205]	The Role Of Masks And Respirator Protection Against Sars-Cov-2	China	LMIC	National	Population Wide
Wax R S [206]	Practical Recommendations For Critical Care And Anesthesiology Teams Caring For Novel Coronavirus (2019-Ncov) Patients	Canada and UK	HIC	Institutional	Healthcare Workers
Wells Patricia [63]	Practical Considerations For The Emergency Delivery Of Babies From Mothers With Confirmed Or Suspected Covid-19	USA	HIC	Institutional	Healthcare Workers
Whiteside T [207]	Redesigning Emergency Department Operations Amidst A Viral Pandemic	USA and Saudi Arabia	HIC	Institutional	Patients and healthcare workers
WHO [208]	Advice on the use of masks for children in the community in the context of COVID-19	Global	HIC and LMIC	Global	Population Wide
WHO [209]	Advice on the use of masks in the context of COVID-19	Global	HIC and LMIC	Global	Population Wide
WHO [211]	Interim Guidance. Infection prevention and control during health care when novel coronavirus (nCoV) infection is suspected	Global	HIC and LMIC	Institutional	Healthcare Workers, Healthcare Managers and Public Health Officials
WHO [210]	Infection prevention and control for the safe management of a dead body in the context of COVID-19	Global	HIC and LMIC	Institutional	Healthcare Workers, Staff of Mortuaries and Public Health Officials
WHO [212]	Considerations for quarantine of contacts of COVID-19 cases	Global	HIC and LMIC	Global	Population Wide
WHO [213]	Home care for patients with suspected or confirmed COVID-19 and management of their contacts	Global	HIC and LMIC	Institutional	Healthcare Workers, Healthcare Managers and Public Health Officials
WHO [214]	Transmission of SARS-CoV-2: implications for infection prevention precautions	Global	HIC and LMIC	Global	Population Wide
WHO [215]	Advice on the use of masks in the community, during home care and in health care settings in the context of the novel coronavirus (2019-nCoV) outbreak: interim guidance, 29 January 2020	Global	HIC and LMIC	Global	Population Wide
WHO [216]	Coronavirus disease (COVID-19) advice for the public	Global	HIC and LMIC	Global	Population Wide
WHO [217]	Clinical management COVID-19	Global	HIC and LMIC	Global	Patients
WHO [218]	Infection prevention and control during health care when novel coronavirus (nCoV) infection is suspected	Global	HIC and LMIC	Global	Healthcare Workers
WHO [219]	Minimum requirements for infection prevention and control programmes	Global	HIC and LMIC	Global	Healthcare Workers and Hospital Visitors
Wickemeyer J L [220]	Evolving Management of COVID-19: A Multi-institutional Otolaryngology Perspective	USA	HIC	Institutional	Healthcare Workers
Wong D H. T [221]	Risk stratification protocol to reduce consumption of personal protective equipment for emergency surgeries during COVID-19 pandemic	China	LMIC	Institutional	Healthcare Workers
Wong J [222]	Preparing for a COVID-19 pandemic: a review of operating room outbreak response measures in a large tertiary hospital in Singapore	Singapore	HIC	Institutional	Healthcare Workers
Workman A D [223]	Airborne Aerosol Generation During Endonasal Procedures in the Era of COVID-19: Risks and Recommendations	USA	HIC	Institutional	Healthcare Workers
Wu H L [224]	Facemask shortage and the novel coronavirus disease (COVID-19) outbreak: Reflections on public health measures	China	LMIC	National	Population Wide
Xu C [225]	Application of refined management in prevention and control of the coronavirus disease 2019 epidemic in non-isolated areas of a general hospital	China	LMIC	Institutional	Healthcare Workers
Yang C Y [226]	Hemodialysis Vascular Access Care During The Covid-19 Pandemic	Taiwan	HIC	Institutional	Patients and healthcare workers
Yao Wenlong [227]	Emergency Tracheal Intubation In 202 Patients With Covid-19 In Wuhan, China: Lessons Learnt And International Expert Recommendations	China	LMIC	Institutional	Patients and healthcare workers
Yao Xiaoyuan [68]	Health protection guideline of passenger transport stations and transportation facilities during COVID-19 outbreak	China	LMIC	National	Transportation workers
Yetmar Z A [228]	Inpatient Care of Patients with COVID-19: A Guide for Hospitalists	USA	HIC	Institutional	Healthcare Workers
Yi-Fong Su [229]	Masks And Medical Care: Two Keys To Taiwan’S Success In Preventing Covid-19 Spread	Taiwan	HIC	National	Population Wide
Ying Bo [69]	Health protection guideline of schools and other educational institutions during COVID-19 outbreak	China	LMIC	National	Students and School Workers
Zhang Liubo [70]	Health protection guideline of mobile cabin hospitals during COVID-19 outbreak	China	LMIC	National	Healthcare Workers
Zhao H M [230]	Recommendations for respiratory rehabilitation in adults with COVID-19	China	LMIC	Institutional	Healthcare Workers
Zhao Yanjie [231]	Radiology department strategies to protect radiologic technologists against COVID19: Experience from Wuhan	China	LMIC	Institutional	Healthcare Workers
Zhou Z [232]	Mask Is The Possible Key For Self-Isolation In Covid-19 Pandemic	China	LMIC	National	Population Wide
Zimmermann M [233]	Approaches to the management of patients in oral and maxillofacial surgery during COVID-19 pandemic	Austria	HIC	Institutional	Healthcare Workers
Zuo M Z [234]	Expert Recommendations For Tracheal Intubation In Critically Ill Patients With Noval Coronavirus Disease 2019	China	LMIC	Institutional	Healthcare Workers

The origin/target of policy documents was analyzed on an income level scale (Table 1) as a growing body of literature suggests that there is significant heterogeneity, both in the direction and magnitude, of association between factors such as socioeconomic status, income inequality and health outcomes. Most policy documents were from high income countries (HIC) such as USA, Italy, Canada and UK. Specifically, 136 policy documents (64.15%) were from HIC [26, 28–33, 37–40, 42–46, 48–58, 60, 61, 63, 71–74, 76, 77, 79–82, 85–94, 99–107, 110, 112, 113, 116, 117, 119, 121–124, 127, 129, 130, 132, 135, 137–139, 143–145, 147–154, 156, 158, 159, 161, 163–165, 167–172, 174–176, 179–182, 184, 185, 188–204, 206, 207, 220, 222, 223, 226, 228, 229, 233], 39 policy documents (18.40%) were from lower middle income countries (LMIC) [23, 24, 27, 35, 36, 47, 62, 64, 65, 67–70, 78, 83, 84, 108, 111, 114, 115, 118, 128, 136, 142, 160, 162, 173, 183, 186, 187, 205, 221, 224, 225, 227, 230–232, 234] and 37 policy documents (17.45%) were from HIC and LMIC [25, 34, 41, 59, 66, 75, 95–98, 109, 120, 125, 126, 131, 133, 134, 140, 141, 146, 155, 157, 166, 177, 178, 208–219] (Table 1).

Mask/PPE policy documents were analyzed for the scale upon which they were implemented (Table 1). The scale was divided into categories: institutional, national and global. Institutional policies were defined as the strategies that were implemented at a unit/institution level such as an ophthalmology center or were targeted towards a specific group that work at an institutional level such as an ophthalmologist. National policies were defined as the strategies that were implemented or intended to be implemented at a national level and included all the population of that nation. Policies from country specific agencies such as CDC, Ministero della Salute etc. were considered national policies. Global policies were defined as the policies that were implemented or intended to be implemented at global level and included everyone across globe. Policies from WHO and any other international agencies were included in this category. Our analysis found that most of the policy documents were implemented at an institutional level. Specifically, 148 policy documents (69.81%) were implemented at institutional level [25, 27–29, 31, 32, 34–38, 40, 42–46, 48, 49, 51, 53, 58, 61–66, 71, 73, 74, 76–78, 80–82, 85, 86, 88–91, 93, 96, 99–110, 112–115, 117, 120–126, 128, 130–139, 142–153, 155–164, 166–168, 171, 173–184, 186, 188–190, 192, 193, 197, 199–204, 206, 207, 210, 211, 213, 220–223, 225–228, 230, 231, 233, 234] whereas 42 policy documents (19.81%) were implemented at national level [23, 24, 26, 30, 33, 39, 47, 50, 52, 55–57, 67–70, 72, 79, 83, 84, 87, 92, 94, 97, 98, 111, 119, 129, 154, 165, 169, 170, 187, 191, 194–196, 198, 205, 224, 229, 232] and only 22 policy documents (10.38%) were implemented at the global level [41, 54, 59, 60, 75, 95, 116, 118, 127, 140, 141, 172, 185, 208, 209, 212, 214–219] (Table 1).

In addition, the target population for the policy documents was also assessed (Table 1). Specifically, 141 policy documents (66.51%) were targeted towards healthcare workers [23, 25–28, 30, 32, 35–42, 44–49, 51, 53, 54, 57, 60–66, 70–73, 76–80, 83, 86–88, 90, 91, 93, 96, 98, 99, 101–105, 107–110, 112, 113, 115–117, 119–127, 130, 133–136, 138, 139, 142–150, 152, 155–164, 166, 167, 169–171, 173, 174, 176–181, 183, 186–190, 193, 194, 197–201, 203, 204, 206, 218, 220–223, 225, 228, 230, 231, 233, 234]; 5 policy documents (2.36%) were targeted towards other workers (mortuary workers, transportation workers, essential workers etc.) [67, 68, 92, 185, 202]; 6 policy documents (2.83%) were targeted towards patients across different disease groups [55, 56, 94, 100, 132, 217]; 30 policy documents (14.15%) were targeted for the general population [24, 33, 50, 52, 59, 75, 84, 89, 95, 111, 114, 118, 129, 140, 141, 154, 165, 172, 191, 196, 205, 208, 209, 212, 214–216, 224, 229, 232] and 30 policy documents (14.15%) had multiple defined target groups [29, 31, 34, 43, 58, 69, 74, 81, 82, 85, 97, 106, 128, 131, 137, 151, 153, 168, 175, 182, 184, 192, 195, 207, 210, 211, 213, 219, 226, 227] (Table 1). Examples of policy documents having multiple target groups were policy documents targeting both patients and healthcare workers; policy documents targeting frontline healthcare workers and hospital administrators and policy documents targeting healthcare workers, staff of mortuaries and public health officials (Table 1).

### Equity incorporation

**Fig. 3:**
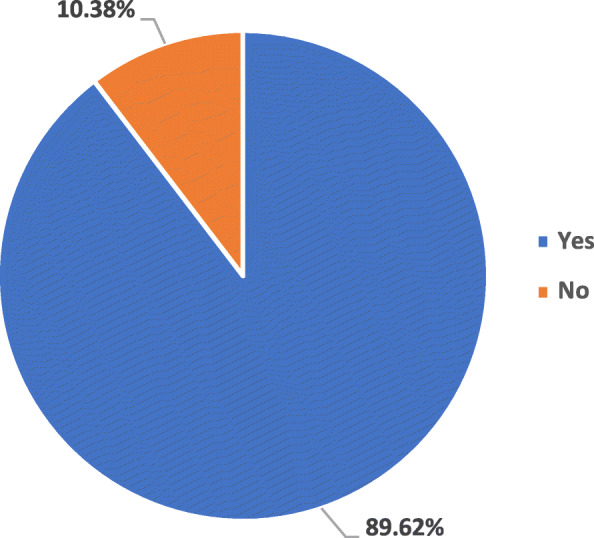
Equity Incorporation (PROGRESS+) Assessment for Included Policy Documents

**Fig. 4:**
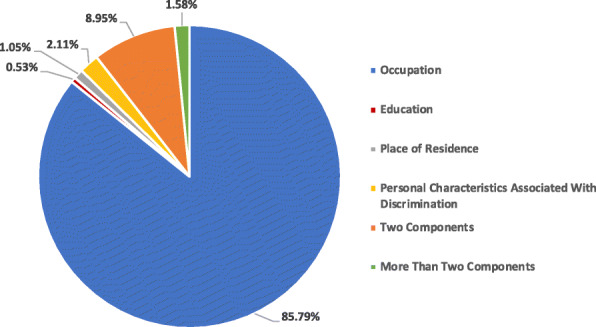
Equity Components (PROGRESS+) For Included Policy Documents

Out of 212 policy documents, 190 policy documents (89.62%) included at least one PROGRESS-plus component [23, 25–49, 51–54, 56–83, 85–88, 90–93, 96–99, 101–117, 119–128, 130, 131, 133–139, 142–164, 166–171, 173–190, 192–195, 197–204, 206–211, 213, 215–223, 225–231, 233, 234] (Fig. 3). The policy documents (*n* = 190) were then categorized in different strata based on the included ‘PROGRESS-Plus” component (Fig. 4). Most of the policy documents focused on “occupation” component of the PROGRESS-plus and included populations at higher risk of contracting COVID-19 such as healthcare workers, essential workers, transportation workers etc. Specifically, 85.79% (*n* = 163) of the included policy documents had “occupation” as an equity component [23, 25–27, 29–32, 34–37, 40, 42–46, 48, 49, 51, 53, 54, 58, 60, 62–68, 70–74, 76–81, 83, 85–88, 90, 91, 93, 96–99, 101–109, 112, 113, 115–117, 119–121, 123–126, 128, 130, 131, 133–136, 138, 139, 142–164, 166–171, 173–184, 186–190, 192–195, 197–204, 206, 207, 210, 211, 213, 215, 217–223, 225–228, 230, 231, 234] followed by personal characteristics associated with discrimination (*n* = 4; 2.11%) [33, 56, 216, 229], place of residence (*n* = 2; 1.05%) [75, 114] and education (n = 1; 0.53%) [69]. Several policy documents had mentioned multiple PROGRESS-Plus components. Specifically, 17 policy documents (8.95%) mentioned two components of PROGRESS-Plus [28, 38, 39, 41, 47, 52, 57, 59, 82, 92, 110, 111, 122, 127, 137, 185, 233] and 3 policy documents (1.58%) mentioned more than two components of the PROGRESS-plus framework [61, 208, 209] (Fig. 4).

**Fig. 5:**
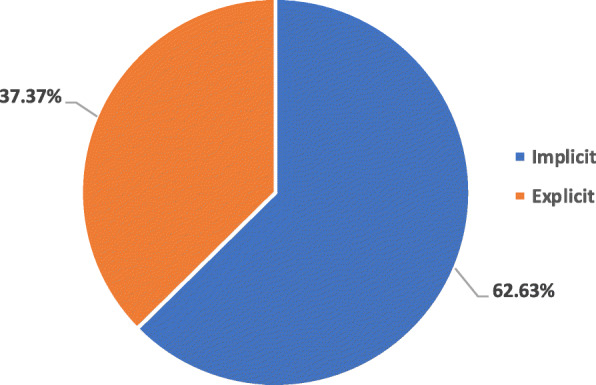
Rationale of Evidence for Justification of Inclusion of PROGRESS-Plus Components in Policy Documents

Second, the strength of rationale to support inclusion of “PROGRESS-plus” factor in a policy document was analyzed. Of all the policy documents having equity component, 71 policy documents (37.37%) explicitly provided the evidence for inclusion of PROGRESS-Plus component [25, 27, 28, 34, 36–38, 41, 42, 49, 58–61, 63, 64, 66, 71, 74–77, 82, 86, 91–93, 104–106, 111, 112, 114, 121, 122, 124, 126, 127, 130, 131, 134–137, 144, 146, 148, 155, 159, 160, 166, 167, 171, 176, 177, 180, 182, 185, 190, 192, 193, 197, 201, 206–208, 226, 227, 231, 233, 234] whereas rest did not provide any rationale for inclusion of PROGRESS-Plus component [23, 26, 29–33, 35, 39, 40, 43–48, 51–54, 56, 57, 62, 65, 67–70, 72, 73, 78–81, 83, 85, 87, 88, 90, 96–99, 101–103, 107–110, 113, 115–117, 119, 120, 123, 125, 128, 133, 138, 139, 142, 143, 145, 147, 149–154, 156–158, 161–164, 168–170, 173–175, 178, 179, 181, 183, 184, 186–189, 194, 195, 198–200, 202–204, 209–211, 213, 215–223, 225, 228–230] (Fig. 5). Among these 71 policy documents, 47 were conducted in HIC [28, 37, 38, 42, 49, 58, 60, 61, 63, 71, 74, 76, 77, 82, 86, 91–93, 104–106, 112, 121, 122, 124, 127, 130, 135, 137, 144, 148, 159, 167, 171, 176, 180, 182, 185, 190, 192, 193, 197, 201, 206, 207, 226, 233], 10 were conducted in LMIC [27, 36, 64, 111, 114, 136, 160, 227, 231, 234] and 14 were conducted in both HIC and LMIC [25, 34, 41, 59, 66, 75, 126, 131, 134, 146, 155, 166, 177, 208]. In addition, most of these policy documents were targeted at workers (*n* = 66; 92.96%) [25, 27, 28, 34, 36–38, 41, 42, 49, 58, 60, 61, 63, 64, 66, 71, 74, 76, 77, 82, 86, 91–93, 104–106, 112, 121, 122, 124, 126, 127, 130, 131, 134–137, 144, 146, 148, 155, 159, 160, 166, 167, 171, 176, 177, 180, 182, 185, 190, 192, 193, 197, 201, 206, 207, 226, 227, 231, 233, 234] and were implemented at institutional level (*n* = 62; 87.32%) [25, 27, 28, 34, 36–38, 42, 49, 58, 61, 63, 64, 66, 71, 74, 76, 77, 82, 86, 91, 93, 104–106, 112, 114, 121, 122, 124, 126, 130, 131, 134–137, 144, 146, 148, 155, 159, 160, 166, 167, 171, 176, 177, 180, 182, 190, 192, 193, 197, 201, 206, 207, 226, 227, 231, 233, 234].

Third, “indication level of equity” in policy documents was assessed. Our analysis found that only 2 policy documents (1.05%) [75, 209] had included terms related to equity and thus were deemed ‘high level’ whereas rest were considered to be of ‘low level’.

### Subgroup analysis

For further analysis, policy documents were subdivided into two groups: policy documents targeted towards workers (*n* = 176; 83.01%) [23, 25–32, 34–49, 51, 53, 54, 57, 58, 60–74, 76–83, 85–88, 90–93, 96–99, 101–110, 112, 113, 115–117, 119–128, 130, 131, 133–139, 142–153, 155–164, 166–171, 173–190, 192–195, 197–204, 206, 207, 210, 211, 213, 218–223, 225–228, 230, 231, 233, 234] and policy documents targeted towards everyone else (*n* = 36; 16.98%) [24, 33, 50, 52, 55, 56, 59, 75, 84, 89, 94, 95, 100, 111, 114, 118, 129, 132, 140, 141, 154, 165, 172, 191, 196, 205, 208, 209, 212, 214–217, 224, 229, 232]. The policy documents were divided as such because workers are usually provided masks/PPE as a part of safety protocol across different working environments such as healthcare setting, shopping centers, transportation centers etc. Among policy documents that were not targeted at workers, the target groups were: “patients” (*n* = 6; 2.80%) [55, 56, 94, 100, 132, 217] and “population wide” (*n* = 30; 14.15%) [24, 33, 50, 52, 59, 75, 84, 89, 95, 111, 114, 118, 129, 140, 141, 154, 165, 172, 191, 196, 205, 208, 209, 212, 214–216, 224, 229, 232].

As expected, all of the policy documents that were targeted towards workers had at least “occupation” component of the PROGRESS-Plus framework. Among policy documents that were not targeted at workers, 16 documents were conducted in HIC [33, 50, 52, 55, 56, 89, 94, 100, 129, 132, 154, 165, 172, 191, 196, 229], 8 were conducted in LMIC [24, 84, 111, 114, 118, 205, 224, 232] and rest were conducted in HIC and LMIC [59, 75, 95, 140, 141, 208, 209, 212, 214–217]. In addition, most of these policy documents were implemented at national level [24, 33, 50, 52, 55, 56, 84, 94, 111, 129, 154, 165, 191, 196, 205, 224, 229, 232]. As opposed to ‘workers related’ policy documents most of these remaining policy documents didn’t have a PROGRESS-Plus equity component rendering them equity limiting. Specifically, 14 policy documents included consideration of a PROGRESS-Plus component [33, 52, 56, 59, 75, 111, 114, 154, 208, 209, 215–217, 229] whereas 22 policy documents didn’t have a PROGRESS-plus component [24, 50, 55, 84, 89, 94, 95, 100, 118, 129, 132, 140, 141, 165, 172, 191, 196, 205, 212, 214, 224, 232]. Specifically, 4 policy documents had ‘personal characteristics associated with discrimination’ as a component [33, 56, 216, 229], 2 policy documents had ‘place of residence’ as a component [75, 114]; 3 policy documents had ‘two components’ [52, 59, 111]; and 2 policy documents had ‘more than two components [208, 209]. Out of 14 policy documents that included a PROGRESS-Plus component [33, 52, 56, 59, 75, 111, 114, 154, 208, 209, 215–217, 229], only 5 policy documents explicitly mentioned the reason to include PROGRESS-plus component [59, 75, 111, 114, 208].

## Discussion

To our knowledge, this is the first systematic review trying to ascertain the extent to which national, regional, institutional and organizational policies reflect equity considerations by focusing specifically on masks and personal protection equipment related policies. We have described the characteristics of the included policy documents including target population and implementation level among others. Most importantly, we have reported the equity factors considered within these policy documents using PROGRESS-plus equity framework. It is important to note that utilization of PROGRESS-plus framework for this research work is context specific and findings are limited to COVID-19 for most part. For instance, population types such as healthcare workers, essential workers are not usually considered vulnerable in society but in the context of COVID-19, increased risk of transmission of COVID-19 puts them at a disadvantage compared to the general population. Thus, findings of the research work should be interpreted within above mentioned scope.

Our review revealed that most of the included policy documents were from HIC. Specifically, we found that 64.15% policy documents were conducted/targeted towards HIC [26, 28–33, 37–40, 42–46, 48–58, 60, 61, 63, 71–74, 76, 77, 79–82, 85–94, 99–107, 110, 112, 113, 116, 117, 119, 121–124, 127, 129, 130, 132, 135, 137–139, 143–145, 147–154, 156, 158, 159, 161, 163–165, 167–172, 174–176, 179–182, 184, 185, 188–204, 206, 207, 220, 222, 223, 226, 228, 229, 233]; 18.40% were conducted/targeted towards LMIC [23, 24, 27, 35, 36, 47, 62, 64, 65, 67–70, 78, 83, 84, 108, 111, 114, 115, 118, 128, 136, 142, 160, 162, 173, 183, 186, 187, 205, 221, 224, 225, 227, 230–232, 234] and 17.45% were conducted/targeted towards both HIC and LMIC countries [25, 34, 41, 59, 66, 75, 95–98, 109, 120, 125, 126, 131, 133, 134, 140, 141, 146, 155, 157, 166, 177, 178, 208–219]. In addition, our analysis found that most of the policy documents were implemented at an institutional level. Specifically, 69.81% of the policy documents were implemented at institutional level [25, 27–29, 31, 32, 34–38, 40, 42–46, 48, 49, 51, 53, 58, 61–66, 71, 73, 74, 76–78, 80–82, 85, 86, 88–91, 93, 96, 99–110, 112–115, 117, 120–126, 128, 130–139, 142–153, 155–164, 166–168, 171, 173–184, 186, 188–190, 192, 193, 197, 199–204, 206, 207, 210, 211, 213, 220–223, 225–228, 230, 231, 233, 234] whereas 19.81% were implemented at national [23, 24, 26, 30, 33, 39, 47, 50, 52, 55–57, 67–70, 72, 79, 83, 84, 87, 92, 94, 97, 98, 111, 119, 129, 154, 165, 169, 170, 187, 191, 194–196, 198, 205, 224, 229, 232] and only 10.38% were implemented at global level [41, 54, 59, 60, 75, 95, 116, 118, 127, 140, 141, 172, 185, 208, 209, 212, 214–219]. These differences might be the result of variations in baseline risk, resources, health, and other system-level factors whether be at institutional or national level that hinder successful implementation of certain policies. For example, guidelines arising from HICs recommending the immediate upscaling of hospital care were not likely to be directly applicable to LMICs such as India because of an already overstretched medical system. In addition, mask mandates enacted in HICs were unlikely to be directly applicable to LMICs due to poverty related issues. Thus, policy makers should consider factors such as socioeconomic status, resource availability, place of residence while designing /implementing the policies. For example, Casola and colleagues [235] highlight that with 12% of US households living under the poverty line, many households may not have been able to purchase an adequate supply of masks for everyone in the household, or have the privilege of isolating at home while an adequate supply is delivered.

Equity assessment analysis revealed that most of the policy documents included only a single PROGRESS-Plus equity component (89.47%) [23, 25–27, 29–37, 40, 42–46, 48, 49, 51, 53, 54, 56, 58, 60, 62–81, 83, 85–88, 90, 91, 93, 96–99, 101–109, 112–117, 119–121, 123–126, 128, 130, 131, 133–136, 138, 139, 142–164, 166–171, 173–184, 186–190, 192–195, 197–204, 206, 207, 210, 211, 213, 215–223, 225–231, 234]. This finding reflects that even if policy documents considered health inequity during the design/implementation, this consideration was very one dimensional in nature. There is significant evidence that determinants of health or health equity related factors coexist across different levels of society and can incur interactive and multiplicative effects among the most disadvantaged subpopulations [236, 237]. People from some racial and ethnic minority groups are more likely to be uninsured than non-Hispanic whites [238]. Healthcare access can also be limited for these groups by many other factors, such as lack of transportation, child care, or ability to take time off of work; communication and language barriers; cultural differences between patients and providers; and historical and current discrimination in healthcare systems. Furthermore, inequities in access to high-quality education for some racial and ethnic minority groups can lead to lower high school completion rates and barriers to college entrance [239]. This may limit future job options and lead to lower paying or less stable jobs. These factors interacting together can not only increase the risk of these subpopulations to contract COVID-19 but also limit their ability to access good medical care. For instance, a poor (socioeconomic status) essential worker (occupation) in LMIC (place of residence) would be at a severe disadvantage of buying PPE/masks and protecting themselves from the transmission of COVID-19, and if contracted would be limited in getting treatment as well. Thus, policy makers should not only consider the presence of different equity related factors but also should consider the possible intersections between them while designing/implementing the policy.

Our review also revealed that very few policy documents acknowledged ‘health equity’ related issues in their text which illustrates that health equity was not a primary factor when these documents were being designed or implemented. Specifically, only 2 policy documents were found to have equity or inequity or health disparities in the aims, objectives or discussion. In addition, our assessment showed that, out of the selected documents, more than 10% of the policy documents [24, 50, 55, 84, 89, 94, 95, 100, 118, 129, 132, 140, 141, 165, 172, 191, 196, 205, 212, 214, 224, 232] had no PROGRESS-plus component mentioned or included in the policy. These findings indicate that significant proportion of policies were enacted with little to no emphasis on the ‘health equity’ mechanisms; which if enacted properly could have further improved the health outcomes for the society as whole.

Lastly, we analyzed the target population for the policies as well. Policy documents were subdivided into two groups: documents targeted towards workers. Our analysis found that majority of policy documents (*n* = 176; 83.01%) were targeted towards workers which was expected as they were at increased risk of contracting COVID-19 when compared to the general population. These targeted policies are usually equity enabling as workers are usually provided masks/PPE as a part of safety protocols across different working environments such as healthcare setting, shopping centers, transportation centers etc. and hence improve COVID-19 related health outcomes. Of the remaining 36 policy documents (16.98%), most were targeted towards whole population (*n* = 30; 14.15%) [24, 33, 50, 52, 59, 75, 84, 89, 95, 111, 114, 118, 129, 140, 141, 154, 165, 172, 191, 196, 205, 208, 209, 212, 214–216, 224, 229, 232]. Most of these policy documents did not include a PROGRESS-Plus equity component rendering them equity limiting for the society. These ‘whole population’ targeted policies should have considered equity as their success in improving COVID-19 related outcomes at a macro scale depends on a number of equity related factors such as education, gender, occupation, place of residence, socioeconomic status among others. Policy makers should identify relevant barriers to successful implementation of population wide policies while formulating such policies.

This study has several limitations that deserve mention. First, our search strategy was limited mainly to biomedical databases such as PubMed, EMBASE, CINAHL and Psycinfo with exceptions of ERIC and ASSIA. The search across a greater number of databases would have led to a substantial increase in the number of retrieved articles thus limiting the feasibility of the process.

In order to address this limitation, we supplemented our search with the documents that were identified by searching the reference list of the documents. Second, it was beyond the remit of this study to assess the potential generalizability of a specific policy, although our analyses of PROGRESS-Plus characteristics indicates that this would have been challenging given that there was little consideration about equity in implementation and impacts within settings, let alone consideration about implementation across different settings. Third, the narrative nature of the findings did not allow us to perform a pooled analysis of any kind (e.g. on the implementation of PPE/masking policies or their impact across different PROGRESS-Plus groups). Although such an analysis would be interesting for future research, this study addressed a different question around the consideration of health equity and inequity in COVID-19 policy and guideline development.

## Conclusion

Policy makers should identify the importance of considering equity via PROGRESS-Plus components while devising guidelines for COVID-19 as these components affect both, risk of acquiring COVID-19 and the COVID-19 associated outcomes. Our review via focus on the masks/PPE policies across the globe highlights that the consideration of equity if present is very, one dimensional in nature. In addition, population wide policies should be carefully designed and implemented after identifying relevant equity related barriers in order to produce better outcomes for the whole society. It is now clear that COVID-19 has disproportionately impacted minoritised and disadvantaged groups and the pandemic has been characterised as killing unequally, with mitigation measures being experienced unequally, and will further impoverish unequally [240]. Our analysis here indicates some of the most important set of policies designed to limit the spread of the pandemic – policies around limiting the pandemic through masking and PPE - were all too frequently devised without equity considerations, and suggests that pandemic response measures were designed from a particular lens (high income country, white, and middle class). In contrast, advancing progress towards equity will generate social, cultural, economic, and environmental wellbeing for the whole society.

## Supplementary Information

**Additional file 1: Table S1.** PRISMA Checklist. **Table S2.** Search Strategy**.**

## Data Availability

All data generated or analyzed during this study are included in this published article and its supplementary information files.
